# Recent advances of enterovirus 71 $3{\rm C}^{{\rm pro}}$ targeting Inhibitors

**DOI:** 10.1186/s12985-020-01430-x

**Published:** 2020-11-11

**Authors:** Rominah Onintsoa Diarimalala, Meichun Hu, Yanhong Wei, Kanghong Hu

**Affiliations:** National 111 Center for Cellular Regulation and Molecular Pharmaceutics, Key Laboratory of Fermentation (Ministry of Education), Hubei Provincial Cooperative Innovation Center of Industrial Fermentation, Hubei Key Laboratory of Industrial Microbiology, Sino-German Biomedical Center, Hubei University of Technology, Wuhan, China

**Keywords:** Enterovirus 71, Enterovirus 71 life cycle, $3{\rm C}^{{\rm pro}}$ functions, $3{\rm C}^{{\rm pro}}$ inhibitors, EV71 drugs screening

## Abstract

With CA16, enterovirus-71 is the causative agent of hand foot and mouth disease (HFMD) which occurs mostly in children under 5 years-old and responsible of several outbreaks since a decade. Most of the time, HFMD is a mild disease but can progress to severe complications such as meningitis, brain stem encephalitis, acute flaccid paralysis (AFP) and even death; EV71 has been identified in all severe cases. Therefore, it is actually one of the most public health issues that threatens children’s life. $3{\rm C}^{{\rm pro}}$ is a protease which plays important functions in EV71 infection. To date, a lot of $3{\rm C}^{{\rm pro}}$ inhibitors have been tested but none of them has been approved yet. Therefore, a drug screening is still an utmost importance in order to treat and/or prevent EV71 infections. This work highlights the EV71 life cycle, $3{\rm C}^{{\rm pro}}$ functions and $3{\rm C}^{{\rm pro}}$ inhibitors recently screened. It permits to well understand all mechanisms about $3{\rm C}^{{\rm pro}}$ and consequently allow further development of drugs targeting $3{\rm C}^{{\rm pro}}$. Thus, this review is helpful for screening of more new $3{\rm C}^{{\rm pro}}$ inhibitors or for designing analogues of well known $3{\rm C}^{{\rm pro}}$ inhibitors in order to improve its antiviral activity.

## Background

Enterovirus 71, belongs to human enterovirus A species, *Picornaviridae* family, was discovered in a patient with central nervous system (CNS), in California, 1969 [1]. In term of structure, EV71 is a non-enveloped virus with a capsid made up of 60 protomers of envelop proteins and contains a single-stranded RNA positive [2, 3]. Each protomer contains four envelop proteins: VP1–VP2–VP3, located in the external part and are exposed to the host antibodies and cell receptors; and VP4 which is completely hidden in the internal part. The RNA genome is small $(7.5\,{\rm kb})$ and constituted by 3 parts: Pl, P2 and P3, flanked by 2 UTRs (non-translated regions) located in $5^{\prime }$ and $3^{\prime }$ [4]. Several outbreaks and fatal cases, caused by this virus, make it a major public health issue mainly in the Asia-Pacific region. Indeed, China has experienced the latest and largest outbreaks with more than 1.7 million cases, 27.000 patients with severe neurological complications and 905 deaths, in 2010 [5]; while a cyclical and seasonal pattern occurs in Sarawak, Japan, Taiwan and Vietnam [6–9]. To manage such infections and epidemics is primordial, and the best way to eradicate this infection is the combination of a valuable vaccine and drugs [10]. Nevertheless, vaccine research has progressed more than drugs discovery because to date there is no approved drug against EV71 while 3 vaccines have completed their clinical trials III and are in following-up stage [11]. For this reason, the treatment is only symptomatic along with public surveillance systems [12]. Many plant extracts and chemical compounds have been discovered as having a potential effects against the virus and might be used as drugs against enterovirus 71 infections but none of them has been approved yet [13]. Thus, the finding of an approved and valuable drug is still an utmost importance. $3{\rm C}^{{\rm pro}}$ represent a valuable target because it has primordial functions in both virulence and virus-host interactions. This review highlights the important functions and recent progress of $3{\rm C}^{{\rm pro}}$ inhibitors and permit to acknowledge that $3{\rm C}^{{\rm pro}}$ is a valuable target for EV71 drug development, which should be deeply investigated.

## Review on EV-71 life cycle

**Fig. 1:**
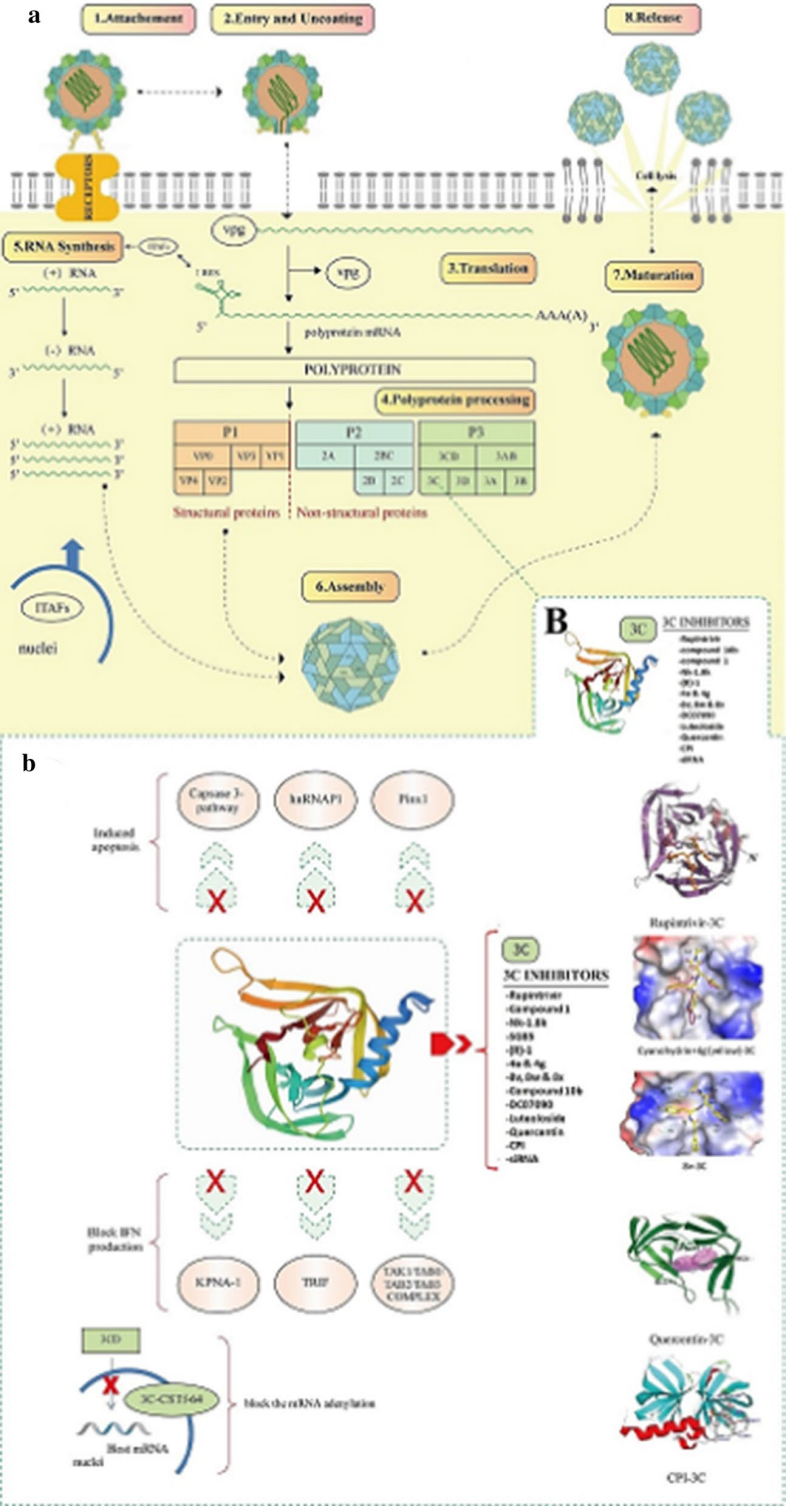
Illustration of EV71 life cycle and virus-host interactions. EV71 replication steps: from attachment to release (**a**). 3C-host proteins interactions are blocked by $3{\rm C}^{{\rm pro}}$ inhibitors (**b**)

The EV71 life cycle goes through an attachment and entry, via a recognition and binding of surface protein to the cell receptors (SCARB2, PSGL-I, Anx2, Heparan Sulfate, Sialylated glycan) [14], to the release of the new virions by cell lysis (Fig. 1a). The mechanism of entry is known as through clathrin-mediated endocytosis (real events remain unclear) but recent investigation has showed that multiple pathways may be used by EV71 to enter the host cells [15, 16]. Then, a series of conformational changes occurs at low pH and let the virus to leave his icosahedral capsid structrure to an A-particle: loss of VP4 and formation of a channel followed by a release of RNA in cell cytoplasm [17]. Once the RNA is located in the cytoplasm, the viral genome, as a positive sense, act as an mRNA, so directly translated into a polyprotein (Pl, P2, P3) of 2193 AA. The polyprotein processing is assured by two main proteins $2{\rm A}^{{\rm pro}}$ and $3{\rm C}^{{\rm pro}}$. Thus, $2{\rm A}^{{\rm pro}}$ and $3{\rm C}^{{\rm pro}}$ cleaved the polyprotein into VP1–VP4 (structural protein) and 2A–2C, 3A–3D (non-structural protein) [18]. When a considerable number of the 11 mature proteins are synthesized, the RNA replication take place after the interactions of IRES-specific-trans-acting factors (ITAFs), which are translocated from the nucleus to the cytoplasm, with the internal ribosome site (IRES) at its stem-loop [19, 20]. A negative-RNA is first synthesis within using the viral genome as template, and then followed by synthesis of numerous positive-strands using in turn the negative-strand as template. RNA-dependent RNA- polymerase (RdRp) or $3{\rm D}^{{\rm po}1}$ is the viral enzyme responsible of the RNA synthesis [18]. Finally, the structural proteins and the genome is encapsidated to form a new virion which is released during lysis of the cell (apoptosis).

## $3{\rm C}^{{\rm pro}}$ functions

In addition to its polyprotein processing activity, the non-structural protein $3{\rm C}$ plays a role in numerous biological mechanisms. Recent discovery of the $3{\rm C}$ crystal structure has permit to identify the sites of its substrat binding affinity (between 2 similar $\beta$-ribbon) and confirmed its cleavage activity of the viral polyprotein but also several host proteins in order to optimize viral replication and spreading [21]. EV71 infection symptoms range from mild to severe diseases which depend on both the viral genetic sequence and the host immune system. In fact, the relationship between $3{\rm C}$ genome sequence and the corresponding clinical symptoms (mild or severe) revealed that the 79th residue is the responsible sequence that leads to severe diseases [22]. Besides, Li et al. [23] have found another residue associated with the virulence of EV71, their finding suggests that the 69th residue is the virulent determinant because a single mutation of the hydrogen bond between Asn69 and Glu71 causes a significant decrease in the EV71 infection. The same result was found during the study of NK-1.8k compound where the substitution of asparagine at 69th residue by serine has decreased the fitness of the virus but on the other hand causes total resistance towards the tested compound. Indeed, the 69th residue plays an important role in $3{\rm C}^{{\rm pro}}$ functions even if it is not directly part of the active site according to the crystal structure [24]. EV71 interacts with the innate immune system through PRRs (Pattern-recognition receptors) such as TLRs which is involved in ${\rm IFN}-{\rm I}$ production, RLRs responsible for detection of RNA virus infection and NLRs which function is to form cytosolic inflammasome [25]. In fact, concomitantly with the virus invasion, different host-immune responses occur such as production of type I interferon (IFN$(\alpha /\beta )$) ; then to escape and to impair the immunity, the virus uses the proteolytic activity of $3{\rm C}^{{\rm pro}}$ by cleaving numerous needed host proteins: KPNA-I in order to suppress the signaling pathway STAT/KPNA-I [26], ${\rm TAK}1/{\rm TAB}1/{\rm TAB}2/{\rm TAB}3$ complex [27], TRIF, shut-off ${\rm IR}3/7$ [28] and consequently block the production of IFN$(\alpha /\beta )$ Likewise, to permit the release and spread of virus progeny, $3{\rm C}$ induced apoptosis of host cells through the capsase-3 pathway [29], cleavage of hnRNPA1 [30] and PinXl [31]. Finally, $3{\rm C}$ is able to enter the nuclei through its precursor $3{\rm CD}$ [32] and cleaves the polyadenylation factor CstF-64. As a result, the host mRNA 3' polyadenylation ,which is essential for its translocation, stability and translation, is shut off [33] (Fig. 1b). Due to such functions, 3C is definitely an excellent target for drug screening.

## $3{\rm C}^{{\rm pro}}$ inhibitors

$3{\rm C}^{{\rm pro}}$ is an important target to block EV71 replication. Indeed, several $3{\rm C}^{{\rm pro}}$ inhibitors have been deeply investigated (Table 1, Fig. 1b)

### Peptidomimetic compounds

- Rupintrivir and analogues: Rupintrivir (AG7088) is probably the well-known $3{\rm C}^{{\rm pro}}$ inhibitors to date. More than being a safe compound for the cells, it is able to bind to the active site of $3{\rm C}^{{\rm pro}}$ [21]. It was firstly identified as $3{\rm C}$ Human Rhinovirus (HRV) inhibitors, later Zhang et al. [34] shown that it also had a strong antiviral activity against EV71 $3{\rm C}^{{\rm pro}}$ in both cell lines and animal models. In fact, AG7088 inhibits the antiviral activity at ${\rm EC}_{50}=0.01\,\upmu {\rm M}$ and protease activity at ${\rm IC}_{50}=2.5\pm 0.5\,\upmu {\rm M}$ with ${\rm CC}_{50}=1000\,\upmu {\rm M}$; in-vivo a low dose of 0.1 mg/kg prevent severe symptoms in suckling mice. Since the discovery of this compound, several analogues have been designed in order to increase its efficiency against EV71 infection [21]. To improve the anti-EV71 activity of rupintrivir, Kuo et al. has designed several inhibitor analogues (compound 1 to $10{\rm b}$) by replacing the P3 group of AG7088 with a series of cinnamoyl derivates. The compound $10{\rm b}$ seemed to be potentially effective against EV71 among all the analogues, with an ${\rm EC}_{50}$ and ${\rm CC}_{50}$ of $0.018\,\upmu {\rm M}$ and $>25\,\upmu {\rm M}$ respectively [35]. Then later Shang et al. [36] replaced the cinnamoyl of compound 1 to 2-chloride-phenylacetyl and noticed that the efficiency of it antiviral activity has been increased twice${\rm IC}_{50}=1.89\pm 0.25\,\upmu {\rm M}$. Another method to further improve rupintrivir action is to combine it with IFN$(\alpha /\beta )$ . In fact, it was proved that rupintrivir and Interferon had an synergistic inhibition against EV71 infection [37].
- NK-1.8k: is a peptidyl aldehyde discovered to have strong anti-viral activity against not only EV71 but also the Enterovirus 68. The mechanism of action is known as the same as rupintrivir which targeted the $3{\rm C}^{{\rm pro}}$ EV71 in dependent-concentration manner. However, structurally, they are different because NK-1.8k is a dipeptide with six-member-ring lactam and rupintrivir, a tripeptide with five-member-ring lactam. Thus, its structure confers to NK-1.8k a better stability and drug features than rupintrivir which is always taken as reference. Indeed, NK-1.8k decrease the viral RNA production at ${\rm EC}_{50}=34.5\, {\rm nM}$. Moreover, it is potent in all the 3 genotypes of EV71 in different cell lines (RD and T293 ${\rm EC}_{50}=0.108\,\upmu {\rm M}$ ; Vero ${\rm EC}_{50}=2.41\,\upmu {\rm M}$) [24]. NK-1.8k represents a new peptidomimetic-compound which might take the place of rupintrivir as an achetype in EV71 drug screening.
- SG85: the $3{\rm C}^{{\rm pro}}$ inhibitors SG85 is a peptidic Michael acceptor compound. It has been tested against Enterovirus 68, EV71, echovirus 11 and various rhinovirus serotypes. However, it was found to be more potent against HRV11 and EV71 with ${\rm EC}_{50}=60\,{\rm nM}$, ${\rm EC}_{50}=180\,{\rm nM}$ respectively [38]. Furthermore, it has screened to have strong antiviral activity against all the 11 EV71 strains with ${\rm EC}_{50}$ between 0.039 and $0.200\,\upmu {\rm M}$ [39]. Deep study of SG85 is needed in order to progress the drug discovery of EV71.
- (R)-1: is proved to be one of the most efficient $3{\rm C}^{{\rm pro}}$ inhibitors screened to date with an ${\rm EC}_{50}=0.088\pm 0.006\,\upmu {\rm M}$. However, the presence of cyanohydrins, which is labile, gives it unstable and toxic properties [40].
- 4e and 4g: are compounds resulted from improvement of (R)-1. In fact, acyl cyanohydrins which make unstable (R)-1 have been replaced by 4-iminooxazolidin-2-one. After a series of test, 4e and 4g were the compound having the most potent antiviral activity with ${\rm EC}_{50}=0.21\pm 0.005$ and $0.033\pm 0.008\,\upmu {\rm M}$ respectively. Moreover, those compounds are safe towards the cell (${\rm CC}_{50}>100\,\upmu {\rm M}$). Thus, they can be used as base for EV71 drug therapy [41].
- 8v, 8w and 8x: are alpha-keto-amid inhibitors against EV71 $3{\rm C}^{{\rm pro}}$. Zeng et al. noticed that the pivotal function of $3{\rm C}^{{\rm pro}}$ makes it the ideal target to fight against EV71 infection. Then, they synthesized several alpha-keto-amids as 3C inhibitors via Passerini reaction. Hence, the compounds 8v, 8w and 8x were exhibiting the most potent antiviral activity against enterovirus 71 with ${\rm EC}_{50}=1.32\pm 0.26, 1.88\pm 0.35\,\,{\hbox {and}}\,\, 1.52\pm 0.31\,\upmu {\rm M}$ respectively. Nevertheless, those compounds should be more improved and studied in order to contribute for EV71 drug discovery which is currently in need [42].

### Non-peptidyl compound: DC07090

Recently identified as novel small potent molecule $3{\rm C}$ inhibitor, it is a non-peptidyl compound designed by docking-based virtual screening and able to bind with $3{\rm C}$ through its binding site and reversible inhibits its protease activity at ${\rm EC}_{50}=22.09\pm 1.07\,\upmu {\rm M}$. Besides, DC07090 has a very low cytotoxicity rate $({\rm CC}_{50}>200\,\upmu {\rm M})$ which makes it an attractive compound for further drug development [43].

### Flavonoids

Flavonoids, originally synthesized by the plants as abiotic stresses: in order to protect themselves against ultraviolet radiation, pathogens and herbivores are a group of natural compounds largely distributed in fruits, vegetables, tea, soy foods and herbs. Most importantly, they have huge therapeutic bioactivities: anti-oxidative, anti-inflammatory and antiviral properties. Researchers used them as a base of drug and dietary supplement in several diseases [44]. They present an attractive therapy for Enterovirus 71 due to their low toxicity towards host cells and their strong antiviral activity.

- Luteoloside: is a flavonoid distributed mainly in *Lonicera japonica*, plant used in traditionnal Chinese medicine, and has got broad activities such as anti-microbial, anti-cancer and antiviral activity against influenza virus, human rhinovirus, coxsackievirus B4 and enterovirus 71. The real mechanisms against EV71 remain unknown and need further deep to elucidate but it is sure that it blocked the pathway at $3{\rm C}$ protease activity stage, ${\rm IC}_{50}=0.36{\rm mM}$ with a selectivity index of 5.3 according to the investigation of Cao et al. Therefore, it is an excellent candidate for drug development [45].
- Ouercetin: is a member of the flavonol subgroup of flavonoid found in many plants, fruits, grains and vegetables with anti-inflammatory, anti-cancer and anti-viral properties. It is probably one of the latest $3{\rm C}$ inhibitor tested. Without toxicity towards the cells, our group’s recent finding reveals that quercentin exhibits a prominent effectivity against the protein $3{\rm C}$ of enterovirus 71 by binding its substrate-binding pocket. Moreover, quercentin seems to have a preventive action. Indeed, cells pre-treated by quercetin present a high survival rate when infected by EV71 virus. Consequently, quercetin may be used both in preventive and in therapeutic application [46]. Therewith, a drug library composing of 1430 FDA approved drugs were previously screened from our laboratory. Interestingly, we found that the compound 3 had significantly anti-EV71 effect among them. Further mechanism study revealed that it targeted viral 3${\rm C}$ protease and block viral replication (unpublished data).
- Diisopropyl Chrysin-7-i1 Phosphate (CPI): is a phosphate ester of chrysin, a natural flavonoid found in many plants. CPI is able to bind in the pocket site of hydrophobic and polar residue of $3{\rm C}$ protease like LEU4- 8, SER-I I I, MET-112. PHE-113 and PRO-115 and inhibits the protease activity at ${\rm EC}_{50}=4.03\,{\rm mM}$. Indeed, $3{\rm C}^{{\rm pro}}$ is unable to cleave human interferon regulator factor 9(IRF9) in the presence of CPI [47].

### siRNA

siRNA is a powerful tool which can be used to target a specific gene in order to suppress it. Small interfering RNA therapeutics has been explored against several human viral infections including Enterovirus due to its specificity and promising effect both in-vitro and in-vivo [48]. Indeed, siRNA recognize, bind and degrade the target mRNA. It is a challenging strategy by the potential risk of mutation, inflammation or immune responses. However, Yang et al. showed that there is any toxicity of the siRNA targeted $3{\rm C}^{{\rm pro}}$ and $3{\rm D}^{{\rm pol}}$ during their investigation. They have designed a novel minicircle vector through $3{\rm C}^{{\rm pro}}$ and $3{\rm D}^{{\rm pol}}$ sequence available in Genbank. In fact, the siRNA did not affect the growth and viability of the cell. Moreover, it has reduced the protein levels to $10.8\pm 6.7\%$, the viral mRNAs to $12.4 \pm 1.75\%$ and the progeny virion production to 15% in infected cells. More importantly, it has protected the infected-suckling mice of a significant weight loss and hind limbs paralysis. Hence, further investigation must be conducted about silencing gene strategy within using $3{\rm C}^{{\rm pro}}$ as target [49].

## Discussion

The unavailable of approved clinical drug makes the finding of a potent compound against EV71 really important. $3{\rm C}^{{\rm pro}}$ is an essential protein for EV71 life cycle and infection, moreover, it has strict subtract and does not have a lot of homologues in mammalian cells [35]. Thus, it is an excellent and attractive target for development of potent drugs. In this review, we summarized several classes of compound recently screened and also rupintrivir which is the drug of reference against $3{\rm C}^{{\rm pro}}$. Actually, rupintrivir and analogues are considered as the most potent $3{\rm C}^{{\rm pro}}$ inhibitors. However, NK-1.8k has almost the same potency and efficiency as rupintrivir (Table 1), and as more stable, it can take the place of rupintrivir as archetype of $3{\rm C}^{{\rm pro}}$ inhibitors. In fact, peptidomimetic compounds represent the most potent class with the minimal effective concentration (180 nM to 2.89 μM, Table 1). It might be due to the fact that they are synthetically designed to fit in the $3{\rm C}^{{\rm pro}}$ active pocket. Nevertheless, flavonoids class, which is composed of active compounds from plants, has satisfactory antiviral activity as well. Indeed, nowadays, the trend of using bioactive compounds as drug candidates is done more and more, because of their broad biological and pharmacological activities, their availability and safety towards the host cells. Besides, the screening of non-peptidyl compound has been tempted but only DC07090 among 50 other compounds has given a satisfactory result [43]. Peptidomimetic compounds might be more potent and interesting than non-peptidyl-compounds. Hence, deep investigation, mainly in an appropriate animal model, should be done for luteoloside, quercentin and CPI which could be approved as EV71 therapy; while more and more peptidomimetic compounds should be designed and/or improved by using the revelation of $3{\rm C}^{{\rm pro}}$ structure as reference. Following the drug screening work, the 69th residue of $3{\rm C}^{{\rm pro}}$, which plays important role in conferring EV71 resistance, could be investigated in order to make sure that the virus will not develop a resistance mutation toward the potent drug as investigated by Wang et al. [24]. Finally, the last recent strategy is the use of RNAi. In fact, there are few investigation about siRNA as therapy against EV71 infection; however, it has been successful against a wide range of viruses: Human immunodeficiency virus, hepatitis B/C virus, Influenza virus [50–53]. Therefore, even if it is a challenging technique, investigating this strategy is worth it.

**Table 1:**
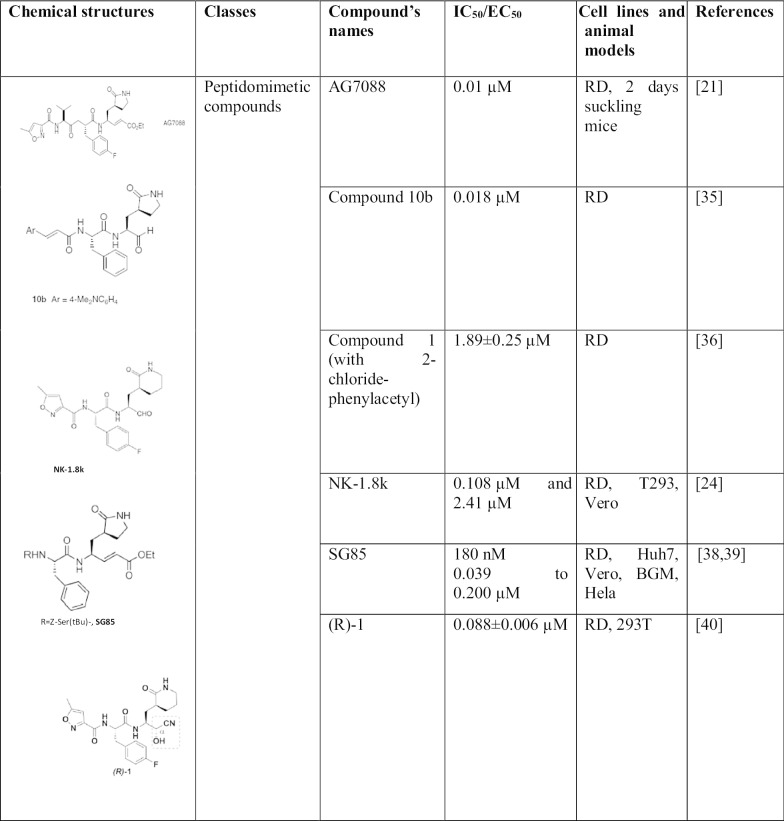

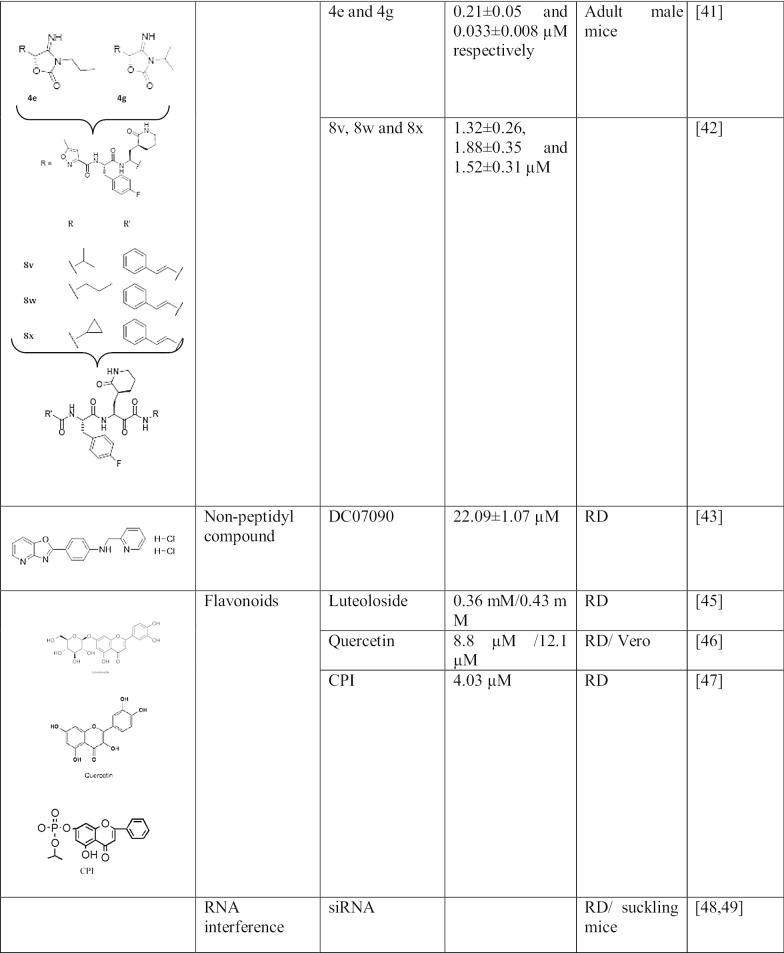
Detailed list and classification of 3C^pro^ inhibitors: chemical structure, classes, effectivity, test in cell lines and animal models

## Conclusion

Coupling an effective vaccine and drugs against Enterovirus 71 is the most prominent manner to eradicate EV71 infection. The prevention will be secure by the vaccine and the treatment by an effective drug. However, the drug progress has not been as developed as for vaccines. In fact, currently only a surveillance is set up to control the disease. EV71 is a threat for children’s life; therefore, the screening of an effective drug is quite indispensable as soon as possible. For that, $3{\rm C}^{{\rm pro}}$ represent an excellent target due to the several key functions that it plays in both virulence and interaction of the virus to the host. More $3{\rm C}^{{\rm pro}}$ inhibitors should be exploited. Besides, as $3{\rm C}^{{\rm pro}}$ and $2{\rm A}^{{\rm pro}}$ play role in early stage of the viral replication through cleaving the EV71 polyprotein, a combination of $2{\rm A}^{{\rm pro}}$ and $3{\rm C}^{{\rm pro}}$ inhibitors in order to act in a synergetic manner may represent a valuable strategy. Indeed, the 3C X-ray structure is already defined so it would promotes further studies of its protease activity inhibitions by a compound. Meanwhile, all drugs screening must be tested in an appropriate animal model which will be compare to the in-vitro screening in order to achieve the goals of using it as treatment against EV71 infections.

## Data Availability

Not applicable.

## Abbreviations

EV71: Enterovirus 71
CA16: Coxsackievirus A16
HFMD: Hand, foot and mouth disease
AFP: Acute flaccid paralysis
3C^pro^: 3C protease
CNS: Central Nervous System
RNA: Ribonucleic acid
UTRs: non-translated regions
SCARB-2: Scavenger receptor class B member 2
PSGL-1: P-selectin glycoprotein ligand-1
Anx2: Annexin-2
2A^pro^: 2A protease
3D^pol^: 3D polymerase
ITAFs: IRES-specific-trans-acting factors
IRES: Internal ribosome site
RdRp: RNA-dependent RNA- polymerase
PRRs: Pattern-recognition receptors
TLRs: Toll-like receptors
IFN-I: Interferon type I
RLRs: RIG-I-like receptors
NLRs: NOD-like receptors
IFN*α*/*β*: Interferon alpha/beta
KPNA-1: Karyopherin subunit Alpha-1
STAT: Signal transducer and activator of transcription
TAK1: Transforming growth factor beta activated kinase
TAB1/2/3: TGF-beta activated kinase 1/2/3
IR3/7: Interferon regulator 3/7
hnRNP: heterogeneous nuclear ribonucleoprotein
PinX1: PIN2 interacting telomerase inhibitor-1
mRNA: messenger Ribonucleic acid
HRV: Human Rhinovirus
EC_50_: 50% Effective Concentration
IC_50_: 50% Inhibition concentration
CC_50_: 50% Cytotoxic Concentration
FDA: Food and Drug Administration
CPI: Chrysin-7-il Phosphate
LEU: Leucine
SER: Serine
MET: Methionine
PHE: Phenylalanine
PRO: Proline
siRNA: Small Intefering RNA
RNAi: RNA interference
RD: Rhabdomyosarcoma
Vero: Verda Reno
BGM: Buffalo green monkey kidney cells
Hela: Henrietta Lack
